# c-REDUCE: Incorporating sequence conservation to detect motifs that correlate with expression

**DOI:** 10.1186/1471-2105-9-506

**Published:** 2008-11-28

**Authors:** Katerina Kechris, Hao Li

**Affiliations:** 1 Department of Biostatistics and Informatics, Colorado School of Public Health, University of Colorado Denver, 4200 East Ninth Avenue, B-119, Denver, CO 80262, USA; 2 Department of Biochemistry and Biophysics, UCSF, 1700 4th Street, San Francisco, CA 94143, USA; 3 Center for Theoretical Biology, Peking University, Beijing 100871, PR China

## Abstract

**Background:**

Computational methods for characterizing novel transcription factor binding sites search for sequence patterns or "motifs" that appear repeatedly in genomic regions of interest. Correlation-based motif finding strategies are used to identify motifs that correlate with expression data and do not rely on promoter sequences from a pre-determined set of genes.

**Results:**

In this work, we describe a method for predicting motifs that combines the correlation-based strategy with phylogenetic footprinting, where motifs are identified by evaluating orthologous sequence regions from multiple species. Our method, c-REDUCE, can account for variability at a motif position inferred from evolutionary information. c-REDUCE has been tested on ChIP-chip data for yeast transcription factors and on gene expression data in *Drosophila*.

**Conclusion:**

Our results indicate that utilizing sequence conservation information in addition to correlation-based methods improves the identification of known motifs.

## Background

An important problem in genome annotation is the identification and characterization of functional elements. These elements include transcription factor binding sites (TFBS), which are short, degenerate sequences that appear frequently in the genome. The interactions between transcription factors (TFs) and their respective binding sites are critical for regulating gene expression. To characterize binding sequences for a TF, computational methods search for sequence patterns or "motifs" that appear repeatedly in genomic regions of interest (for a recent review, see [1]).

For many motif-finding methods, it is necessary to input upstream sequences from a set of genes (e.g., genes that have been identified as co-expressed from a microarray gene expression analysis), with the assumption that a common motif is shared by the sequences (e.g., [2,3]). However, upstream sequences of genes included in this set may not have an occurrence of the same motif, or genes that have the occurrence of the motif in their upstream sequence may not be identified in the co-expressed set. To address these weaknesses, correlation-based motif finding methods [4] have been developed that do not rely on a pre-determined set of genes either based on co-expression (e.g., [2,3]) or over-representation of motifs as in [5]. Using all genes from a single experiment, oligos in a specified length range are enumerated in their upstream sequence and tested for significant correlation with expression values or genome-wide location measurements for a particular TF. The correlation-based motif finding approach was introduced in the "Regulatory Element Detection Using Correlation with Expression" (REDUCE) software [4] using a linear regression framework and has since been adapted in several ways including the use of scores to motifs instead of oligo counts [6], probabilistic representations of motifs [7], binary indicators for word occurrences [8] and flexible non-linear regression functions [9,10].

An alternative motif-finding strategy, relying on the availability of complete genomes from related species, has made it possible to search for putative TFBS in evolutionarily conserved sequences. It has been shown that for closely related species, where reasonable alignment of the orthologous promoter sequences can be achieved, the binding sites for many TFs are evolutionarily conserved. Different computational methods have been developed that vary in the number and diversity of species investigated, in search strategies, i.e. genome-wide (e.g., [11,12]) versus gene sets (e.g., [13]), in whether they use known transcription factors motifs (e.g., [14]) or predict motifs *de novo *(e.g., [15]), in how they integrate inter-species conservation with intra-species conservation (e.g., [16]), in whether the alignment of the motif occurrences across species is required (e.g., [17]) and in whether global alignments in orthologous sequences are necessary [18].

In summary, there are numerous motif finding methods that fall into several different classes, including those reviewed that are correlation or sequence-conservation based. Because of their successes individually, in this work, we describe a new method for predicting motifs that combines these two strategies.

Due to the variability in TF-DNA interactions, TFBS are characterized by motifs containing degenerate positions. For example, the second position in the consensus TFBS for the yeast transcription factor OPI1 (GRTTCGA) can be A or G, which is denoted by the IUPAC symbol R. At a functional TFBS, the possible substitutions at a position may be observed in aligned sequences from multiple species. For example, an OPI1 functional site may be fully conserved across species (as GATTCGA or GGTTCGA) or exhibit A or G at the second position for different species.

To search for degenerate motifs, we have developed an adaptation of the correlation-based algorithm REDUCE [4] called conservation-REDUCE (c-REDUCE). In c-REDUCE, a multiple species alignment is generated and then translated into a consensus pattern using degenerate nucleotide symbols that capture the variation at each position across species. All oligos, including those with degenerate symbols, are then evaluated for significant correlation. By using multiple species data, we can identify motifs that may be missed by REDUCE, which only examines sequences from a single species and requires exactly the same oligo in different sequences.

An alternative method for identifying degenerate motifs is fast-REDUCE (f-REDUCE) [19], which was developed for single species data and identifies degenerate motifs through an enumerative approach. However, enumeration of degenerate motifs can become very costly as the length of the motif and number of degenerate positions increases. In contrast, c-REDUCE reduces the search space of degenerate motifs by taking into account the variability at a position inferred from evolutionary information.

In summary, c-REDUCE benefits from the use of conservation in two ways. First, it predicts degenerate motifs, but reduces the search space by only focusing on naturally occurring degeneracies that appear across multiple species. Second, by examining sequences from multiple species, it will discount chance matches of a motif in a single species if it the match has a highly degenerate consensus sequence in the multiple species alignment. The degeneracy of the consensus, reflecting random mutations in other species, makes a functional TFBS at that position less likely. To predict transcription factor binding site motifs, our method is evaluated on ChIP-chip (chromatin immunoprecipitation on microarray) data in yeast and gene expression data in *Drosophila*. We find that the conservation and correlation-based approaches perform better in combination than they do individually.

## Results

### c-REDUCE applied to yeast data

c-REDUCE was first applied to the 78 genome-wide location data sets of 37 TFs where six other methods failed to identify the motif specified in the literature for that TF [20]. These six alternative methods were applied to sets of sequences that were determined to be significantly enriched for TF binding. Two of the six methods also incorporated sequence conservation. In comparison, c-REDUCE uses upstream sequences from the entire set of genes AND incorporates conservation information. The results for both c-REDUCE and f-REDUCE (degenerate motifs but without conservation) are displayed in Tables 1 and 2. For 18 of the 37 transcription factors, c-REDUCE identified the specified motif in at least one of the conditions, while f-REDUCE discovered the correct motif for 10 transcription factors. In many cases, both programs were successful, but f-REDUCE often discovered a shorter or more degenerate motif than c-REDUCE. Both programs are not suitable for finding long motifs with dimer patterns. Therefore, some of the missed cases were for TFs such as GAL80, with the motif CGGn(11)CCG.

**Table 1:** Summary of results comparing c-REDUCE and f-REDUCE oligo predictions for ChIP-chip yeast data (part 1).

**TF**	**Motif**	**Condition**	**c-REDUCE**	**f-REDUCE**
**ADR1**	GGRGK/MCYCC	**YPD**		
		**HEAT**		
		**SM**		

**ARR1**	TTACTAA/TTAGTAA	**YPD**		4: WCHAA 12.2
		**H2O2Hi**		

**ASH1**	YTGACT/AGTCAR	**YPD**		
		**BUT14**		

**DAL80**	GATAA/TTATC	**YPD**	4: TTAKM 2.1	
		**RAPA**		

**DAL81**	AAAAGCCGCGGGCGGATT/	**YPD**		
	AATCCCGCCCGCGGCTTTT	**RAPA**		
		**SM**	2: RCGGC 13.5	
			3: AAAAR 9.0	

**GAL80**	CGGN(11)CCG	**YPD**		

**GCR1**	GGCTCCWC/	**YPD**		
	GWGGAGCC			

**GZF3**	GATAAGATAAG/	**YPD**		
	CTTATCTTATC	**RAPA**		

**HAC1**	KGMCAGCGTGTC/	**YPD**		3: AYACK 4.4
	GACACGCTGKCM			

**HAP2**	CCAAT/ATTGG	**YPD**	1: ATTGGY 3.0	5: CCAATCA 6.7
		**RAPA**	1: CCAATCA 17.2	
			4: ATTGGY 3.9	

**HAP3**	CCAAT/ATTGG	**YPD**	2: YCAAD 4.8	
**HAP5**	CCAAT/ATTGG	**YPD**	1: ATTGGY 7.6	
			2: YCAAD 4.1	
		**SM**	3: CCAATCA 5.0	

**MAC1**	GAGCAAA/TTTGCTC	**YPD**	1: GAGCAAA 26.6	4: GAGCAAA 3.5
			2: TTTGCTC 16.4	
		**H2O2Hi**	1: GAGCAAA 12.3	
			2: TTTGCTC 6.4	

**MET31**	AAACTGTGG/	**YPD**	4: CACAGT 2.7	
	CCACAGTTT	**SM**		

**MET32**	AAACTGTGG/	**YPD**	1: CACAGTT 10.2	
	CCACAGTTT	**SM**	1: CACAGTT 32.3	
			4: AACTGTG 8.3	

**MOT3**	YAGGYA/TRCCTR	**YPD**		
		**H2O2Lo**		
		**H2O2Hi**		
		**SM**		

**MSN4**	MAGGGG/CCCCTK	**YPD**		
		**RAPA**		
		**H2O2Lo**		
		**H2O2Hi**	1: CCCCT 16.7	
			3: AAGGGG 4.1	

**OPI1**	TCGAAYC/GRTTCGA	**YPD**	2: GTTCGA 6.2	

**PDR3**	TCCGCGGA/TCCGCGGA	**YPD**		

The 37 transcription factor (TF) motifs not discovered by the methods applied in [20] are listed in Tables 1 and 2. Only exact matches to the motifs (see Methods) are considered. The first and second columns list the transcription factor and known motif given in the Supplementary Table 3 file from [20]. The third column lists the environmental conditions examined (YPD: Rich medium, HEAT: Elevated temperature, SM: Amino acid starvation, H2O2Hi: Highly hyperoxic, H2O2Lo: Moderately hyperoxic, BUT14: Filamentation inducing, RAPA: Nutrient deprived and GAL: Galatose medium). The fourth and fifth columns list the results for c-REDUCE and f-REDUCE respectively. For example, "1: ATTGGY 3.0" for HAP2, indicates that the oligo ATTGGY was the first predicted oligo with a -log_10_(p-value) of 3.0 under the YPD condition. The degenerate symbols are R = (A, G), Y = (C, T), M = (A, C), K = (G, T), S = (C, G), W = (A, T), B = (C, G, T), D = (A, G, T), H = (A, C, T), V = (A, C, G) and N = (A, C, G, T).

**Table 2:** Summary of results comparing c-REDUCE and f-REDUCE oligo predictions for ChIP-chip yeast data (part 2).

**TF**	**Motif**	**Condition**	**c-REDUCE**	**f-REDUCE**
**PUT3**	CGGN(11)CCG	**YPD**		1: GVVCG 35.2
				2: CVCVG 15.1
		**H2O2Lo**		
		**SM**		

**RGT1**	CGGANNA/TNNTCCG	**YPD**		2: CCHCV 10.8
		**GAL**		

**RIM101**	TCGGAAG/CTTCCGA	**YPD**		
		**H2O2Lo**		
		**H2O2Hi**		

**RLM1**	CTAWWWWTAG/	**YPD**	1: TATTT 11.8	1: DTTWA 24.4
	CTAWWWWTAG		2: AARAW 7.5	2: AAVHTA 10.9
			3: RAWTT 5.7	
			5: TTTYY 4.0	
		**BUT14**		

**ROX1**	YSYATTGTT/AACAATRSR	**YPD**		
		**H2O2Lo**		
		**H2O2Hi**		

**RPH1**	CCCCTTAAGG/	**YPD**		
	CCTTAAGGGG	**H2O2Lo**		
		**H2O2Hi**	1: CCCCT 16.1	
			3: AAGGGG 3.8	
		**SM**		

**RTG3**	GGTCAC/GTGACC	**YPD**		
		**H2O2LO**		
		**H2O2HI**		
		**RAPA**	4: RTGAC 2.6	
		**SM**		

**SKO1**	ACGTCA/TGACGT	**YPD**	2: ACGTCAT 5.1	1: VCGBC 19.0
				4: ATGACGT 3.1

**SMP1**	ACTACTAWWWWTAG/	**YPD**	3: TTAATAG 6.3	2: TTTHA 9.4
	CTAWWWWTAGTAGT			

**STP1**	RCGGCNNNRCGGC/	**YPD**		
	GCCGYNNNNGCCGY	**SM**	2: CGGCAY 3.0	
			4: TMAGR 2.7	
			5: RCGGY 2.2	

**SWI5**	KGCTGR/YCAGCM	**YPD**	3: TGGCTGG 2.5	2: CBDGC 4.7
				3: GKSTG 1.4

**UGA3**	CCGNNNNCGG	**YPD**		
		**RAPA**		
		**SM**		

**XBP1**	CTTCGAG/CTCGAAG	**YPD**		
		**H2O2Lo**	1: TCGAG 27.9	
			5: TCGAR 7.3	

**YAP3**	TTACTAA/TTAGTAA	**YPD**		
		**H2O2Hi**		

**YAP5**	TTACTAA/TTAGTAA	**YPD**		
		**H2O2Hi**		

**YAP6**	TTACTAA/TTAGTAA	**YPD**		
		**H2O2Lo**		
		**H2O2Hi**		

**YHP1**	TAATTG/CAATTA	**YPD**		

**YOX1**	YAATA/TATTR	**YPD**		

See Table 1 for details.

We also ran c-REDUCE on the complete set of 81 TFs given in [20]. We separated the complete set into the 44 TFs with motifs that were recovered by one of the programs applied by [20] (labeled "Recovered") and the 37 from above that were not recovered by any of their programs (labeled "Not-recovered") (See Additional File 1: Supporting Table 1). While we were unable to find some of the Recovered motifs, c-REDUCE, using exact matches, performed much better on the Not-recovered set for a total correct prediction rate of ~65% compared to ~54% in Harbison *et al. *[20] (Table 3). We also relaxed our matching criteria to allow for one mismatch or one shifted match (1 MM/S). For example, in Harbison *et al. *[20], although there was one mismatch, the predicted motif "CACATGC" was considered a successful prediction for the known INO2 motif "ATTTCACATC". We discovered the motif "TCACATG", very similar to their predicted "CACATGC", but because of the last position "G" it was not considered an exact match. Therefore, relaxing our criteria to allow for one mismatch or one shifted position, we were able to improve our correct prediction rate to 79% (Table 3).

**Table 3:** Summary of results on ChIP-chip yeast data.

	Harbison *et al.*[20]	c-REDUCE (exact)	c-REDUCE (1 MM/S)
Recovered (44)	44	38	40
Not-recovered (37)	0	18	24

Total (81)	44 (~54%)	53 (~65%)	64 (~79%)

For each transcription factor motif, c-REDUCE results are listed for the complete Harbison *et al. *[20] data using either exact matches or at most one mismatch or shifted match "1 MM/S" (see Methods).

### Comparisons with other programs

We compared c-REDUCE with four other programs that also use multiple species data for motif prediction on the more difficult "Not-recovered" set from Harbison *et al. *[20]. All methods we evaluated, Phylocon [21], Converge [21], Phylogibbs [22] and Tree Gibbs Sampler [17,23], are designed to be applied to orthologous sequences from a subset of all genes. PhyloGibbs and Tree Gibbs Sampler also incorporate the phylogenetic relationships among the species into their search. Although sequence conservation is incorporated into these methods, they differ from c-REDUCE in that they use a sequence set approach, where only sequences with the most significant TF binding enrichment are used rather than all sequences.

In the PhyloGibbs program [22], there were a total of 21 TF data sets in the "Not-recovered" list where we could compare our results and in those cases, both programs made 16 correct predictions, although not always for the same TF motif (Table 4, Additional File 1: Supporting Table 2). In comparison to the method of Tree Gibbs Sampler [17,23], which was applied to a subset of the 15 "Not-recovered" TFs, c-REDUCE with 1 MM/S made more correct predictions and less false positive predictions (Table 4, Additional File 1: Supporting Table 3). Finally, for Converge and PhyloCon [21] results for almost all of the "Not-recovered" cases were reported in their "Additional File 2". c-REDUCE was able to correctly predict ~65% of the motifs, while PhyloCon and Converge predicted ~26% and ~41% respectively (Table 4, Additional File 1: Supporting Table 4). Only when their predictions were combined did these two programs have more similar accuracy to c-REDUCE.

**Table 4:** Summary of comparisons of c-REDUCE (1 MM/S) with other programs on 37 "not-recovered" cases.

	c-REDUCE	PhyloGibbs [22]	
		
Total (21)	16	16	
Total (15)	c-REDUCE		Tree Gibbs Sampler [17]	

True Positives	11		8	

False Positives	3/14 (21.4%)		5/13 (38.5%)	

	c-REDUCE	PhyloCon [21]	Converge [21]	PhyloCon & Converge [21]

Total (35)	22	9	14	20

The sub-tables list comparisons between c-REDUCE and several other methods. The total number of transcription factor datasets evaluated (Total) is not the same in each sub-table because results are not always reported for the complete 37 "Not-recovered" set. For Tree Gibbs Sampler, the authors report all motif predictions and the false positive rates can be compared with c-REDUCE. For that sub-table, "True positives" indicates the number of correct predictions and "False positives" indicates the number of incorrect predictions out of all predictions.

There are some caveats regarding the comparisons between methods.  We use reported successes by the authors since motif predictions were not always provided and the evaluation criteria, typically for predicted position weight matrices, were usually quite different from those for the c-REDUCE oligo predictions. For Tree Gibbs Sampler [17,23], we could make a more direct comparison because the authors provided all consensus motifs for their predictions and their reported evaluation criteria could be used to compare the c-REDUCE oligos with the known motif. However, the Tree Gibbs Sampler evaluation may be at a disadvantage to c-REDUCE because degenerate symbols were not used to construct their consensus motifs. Because of this, the Tree Gibbs Sampler incorrect predictions were manually checked with a more relaxed criteria allowing for more mismatches, but the results in Table 4 did not change.

### Application to insect data

To investigate the performance of c-REDUCE on more diverse species, we applied our method to insect data. To predict the binding site motif for the *Drosophila *(fruit fly) transcription factor Dorsal, we applied c-REDUCE to data from a microarray study on Dorsal targets [24] and 5 kilobase upstream sequences from four insect species (See Methods). Dorsal is important for the initiation of tissue differentiation in the early embryo and many of its target genes are sequence-specific transcription factors. The experiment in [24] determined genome-wide expression levels comparing mutant embryos that contain no Dorsal protein (none) or uniformly high levels (high) or low levels (low) of Dorsal throughout the embryo. Known Dorsal binding sites are represented by the consensus sequences GGGWWWWCCM or GGGWDWWWCCM [25]. Table 5 summarizes the results of running c-REDUCE on the Dorsal data. For all three comparisons (high vs. none, high vs. low, low vs. none) the top predicted oligo matched the consensus Dorsal binding sites using exact matches. REDUCE, without the conservation information, was unable to predict this binding site, although it did predict the motif ACCCC for high vs. none (-log_10 _p-value = 3.15) and high vs. none (-log_10 _p-value = 4.71) but it is not an exact match to the known motif.

**Table 5:** c-REDUCE results on Dorsal expression study.

Experiment	Oligo	Rank	-log_10_(p-value)
high vs none	ATRT**CCY**	1	20.1
	**KGR**AGAT	2	8.4
	**GG**TRKT	3	6.5

high vs low	ATRT**CCY**	1	43.1
	**TGG**TRKT	2	33.7

low vs none	**GG**AAAR**S**	1	5.5
	**KGR**AGAT	4	2.7

The first column shows the pair-wise mutant comparisons (see text). Columns 2–4 list predicted oligos that match the Dorsal motif, their rank and -log_10_(p-value) respectively. Positions in bold indicate matches to the flanking GGG/CCC or KGG/CCM part of the consensus Dorsal motifs (GGGWWWWCCM or GGGWDWWWCCM [25]).

## Discussion

c-REDUCE is a straightforward adaptation of the REDUCE software. It uses the same algorithm but requires consensus sequences from multi-species alignments and an initial enumeration of oligos to remove those that have more than two degenerate symbols from the search process. Because it takes advantage of the speed in REDUCE, it is well suited for searching long sequences. In contrast, programs such as Tree Gibbs Sampler have been limited to testing at most 1 kilobase regions. Recently, sequence conservation data was used to enhance MatrixREDUCE [7,26], an adaptation of the REDUCE algorithm, but not for the purpose of *de novo *motif finding, which is the focus here.

c-REDUCE in its current form has some limitations. Some transcription factors motifs were not predicted by c-REDUCE, possibly due to dimer patterns that are not well characterized by this method, weak evidence for this motif in the literature, or low quality of the genome-wide location data. c-REDUCE is not well suited for finding very degenerate motifs; we only allow 2 degenerate symbols total. It is also not well suited for searching very long motifs (> 10 base pairs) because of the small sample size associated with matches to the longer motifs. c-REDUCE also has some limitations in particular for higher eukaryotes. It requires global alignments of the sequences, expects aligned binding sites within the global alignment, and does not explicitly model *cis*-regulatory modules. Despite these limitations, it was successfully applied to a difficult insect data set.

The yeast data set is a common benchmark test set and consists of *Saccharomyces sensu stricto *species which are only separated by ~20 MYA [27]. For a more challenging prediction problem, we applied c-REDUCE to insect data with species separated by ~250 MYA (between *Drosophila *and mosquito) [28]. In higher eukaryotes, TFBS are often dispersed in long intergenic regions so it is important to consider a longer upstream sequence region. However, TFBS are typically less than 20 base pairs, so increasing the sequence search space decreases the signal to noise ratio making the search problem more difficult. Despite these challenges, c-REDUCE correctly predicted the Dorsal motif in all three gene expression comparison experiments.

## Conclusion

c-REDUCE, which relies on sequence conservation and a correlation-based strategy across all gene upstream sequences, shows improved performance on a comprehensive genome-wide location dataset for yeast. Our comparisons to f-REDUCE, which does not use sequence conservation, and to several other programs that use conservation but only on sequences from gene subsets, indicate that the combination of these two approaches yields more predictive power. c-REDUCE can be used to find degenerate motifs but instead of relying on exhaustive searches with degenerate symbols, which can quickly become intractable, it limits the search by taking advantage of evolutionary information and discounts false occurrences of motifs that are not evolutionarily conserved.

## Methods

### Yeast Data

Genome-wide location analysis results of 203 transcription factors in *Saccharomyces cerevisiae *under several environmental conditions were taken from Harbison *et al. *[20]. For each experiment, there is a transcription factor binding enrichment value for ~5000 gene promoters. For each gene promoter, orthologs from five yeast species (*S. cerevisiae, S. paradoxus, S. mikatae, S. kudriavzevii *and *S. bayanus*) were obtained from [11,12] and aligned using ClustalW [29]. The average alignment length is ~800 base pairs.

### Insect Data

Expression data to identify Dorsal targets using the Affymetrix GeneChip Drosophila Genome Array DrosGenome1 [30] were obtained from GEO ([31]) with GEO identifier Series GSE86. We obtained a four species insect genome alignment from UCSC Genome Browser (Jan. 2003 [32]). The alignment contained three *Drosophila *species, *D. melanogaster *(BDGP Release 3)*, D. yakuba *(WUSTL Release 1.0), and *D. pseudoobscura *(HGSC Freeze 1), and mosquito *Anopheles gambiae *(Release IAGP v.MOZ2). The alignments provided by UCSC are separated into blocks, which we concatenated by the symbol "N". A 5000 base upstream region for each gene listed in FlyBase (BDGP Release 3 [33]) was extracted from the alignment with overlapping regions from upstream genes removed. The array consists of 12,782 probes for annotated genes, of which 10,572 have upstream sequence alignments greater than 100 base pairs.

### Consensus Sequence

We created a consensus sequence for multiple species alignments using the following procedure. At each position, one of the IUPAC symbols for the four bases (A, C, G, T) or degenerate symbols representing multiple bases (e.g., W = A or T), is used for the observed nucleotides (see examples in Table 6, positions 1 and 2). If less than half of the sequences have "N"s or gaps ("-"), we ignore those symbols. But if the majority of symbols at a position are "N"s, "-"'s or both, we use "N", "-" or "-" respectively (positions 3–5) For some sequences, if a "N' or gap appears and there are only two sequences in the alignment including the reference genome *S. cerevisiae *(or *D. melanogaster*), we use the symbol observed in *S. cerevisiae *(or *D. melanogaster*) (Position 6–7).

**Table 6:** Examples of consensus construction.

	Position						
	
	1	2	3	4	5	6	7
Scer	A	A	G	C	A	A	-
Spar	A	A	G	N	N	-	T
Smik	A	A	-	N	N	*	*
Skud	A	T	-	N	-	*	*
Sbay	A	T	-	N	-	*	*

Consensus	**A**	**W**	**-**	**N**	**-**	**A**	**-**

Sequences (rows) are from the yeast species, *S. cerevisiae *(Scer), *S. paradoxus *(Spar), *S. mikatae *(Smik), *S. kudriavzevii *(Skud) and *S. bayanus *(Sbay). An asterisk indicates that there are no sequences from that species that could be aligned.

c-REDUCE results were obtained by running the REDUCE program, provided by the authors [4], on the multiple species consensus sequences. The program evaluates all oligos, including those with degenerate symbols (in c-REDUCE), for significant correlation across all genes between the counts of the oligo in a gene promoter and the experimental values (*e.g.*, gene expression or TF binding enrichment) for that gene. We examine oligos 5–7 base pairs long for both c-REDUCE and f-REDUCE. Because of the large number of oligos with degenerate symbols, we only test oligos with at least 10 counts and at most two degenerate symbols in c-REDUCE. Methods for finding multiple oligos and for determining statistical significance are described in [4]. Any oligos with gaps due to the alignment were not considered in the analysis.

For oligos with degenerate symbols in c-REDUCE, we considered two options for degenerate oligo counting in our sequences illustrated by the following examples: 1) "W" is counted only when "A" or "T" are in the global alignment; 2) "W" is counted when "A", "T" or "W" are in the global alignment. We did not consider a third option of "W" being counted when "D", and "H" are in the global alignment, since "W" would only characterize a subset of the observed nucleotides. However, in #2, "D" is counted when "A", "T", "G", "K", "R", "W", or "D" occur. We found that option #2, although intuitively more correct, was much slower on the yeast data and did not dramatically improve the results based on option #1.

f-REDUCE results were provided by the authors [19]. To make a fair comparison, f-REDUCE was applied with the same oligo options as c-REDUCE (5–7 length oligos and allowing for 2 and 3-fold degenerate symbols). Although these options are different than the options reported in [19] and produced different predictions in some cases, the results in Tables 1 and 2 and "Supplementary Table 1" from [19] are still consistent qualitatively.

### Motif Evaluation

We took the top 5 significant oligos positively correlated with transcription factor enrichment or expression from c-REDUCE and f-REDUCE based on a forward selection process (p-value < .01). Then, using a script written in perl we compared these oligos with the consensus motif (and its reverse complement) found in the literature [17,20-22,25] for each TF. Degenerate IUPAC symbols are expanded for matching. For example, the predicted oligo YCAAD would be considered a match to the HAP3 motif (CCAAT/ATTGG) since it can be expanded into the oligos which include CCAAT. If there was no exact match to one of the top 5 predicted oligos, we then considered at most 1 mis-match (e.g., the predicted oligo TCACATG matches the motif ATTTCACATC) or one shifted position (e.g., the predicted oligo RCATTC is shifted one position from the motif CATTCY). When comparing an oligo with a motif at a position where both have a degenerate symbol, we checked if there are any nucleotide overlaps. For example, the predicted oligo SAATA would match the motif for YOX1 YAATA because S = (C, G) and Y = (C, T) overlap at the C nucleotide.

### Comparison with Other Software

We compared c-REDUCE predicted oligos with the list of motifs for 81 TFs in "Supplementary Table 3" from Harbison *et al. *2004 [20], list of 35 "Not-recovered" motifs (see definition above) in MacIsaac *et al.*, 2006 [21] (Converge/PhyloCon), list of 15 "Not-recovered" motifs in Li and Wong, 2005 [17] (Tree Gibbs Sampler) and list of 21 "Not-recovered" motifs in Siddharthan *et al.*, 2005 [22] (PhyloGibbs).

MacIsaac *et al.*, 2006 [21] and Li and Wong, 2005 [17] only use a 4 species alignment. Therefore, to compare with these methods we removed *S. kudriavzevii *and *S. paradoxus *respectively to construct our consensus sequence. For comparison with Li and Wong, 2005 [17], we used the curated list of "Not-recovered" motifs found in "Supporting Table 4" of their paper and their definition of a match: "The criterion for matching with TRANSFAC motifs is that there should be at most one mismatch in the orange regions. Those orange regions must be continuous except the ambiguous positions ... The length of the orange parts must be at least 6 unless the motif is shorter than 6." For comparison with PhyloGibbs [22], we used the "Not-recovered" TFs listed in their "Table 1" and the motifs they extracted from . For comparison with MacIsaac *et al. *2006 [21], we used the "Not-recovered" TFs and corresponding motifs from their "Additional File 2".

## Abbreviations

TFBS: transcription factor binding site(s); ChIP-chip: chromatin immunoprecipitation on microarray; REDUCE: regulatory element detection using correlation with expression; f-REDUCE: fast-REDUCE; c-REDUCE: conservation-REDUCE; 1 MM/S: one mismatch or one shifted match.

## Authors' contributions

KK conceived and implemented the method, ran all test sets and drafted the manuscript. HL supervised method development and data analysis. Both authors read and approved the final manuscript.

## Supplementary Material

Additional file 1 **This file contains 4 supporting tables listed below**. Supporting Table 1: Complete set of c-REDUCE results on yeast data. Supporting Table 2: Comparison of c-REDUCE with PhyloGibbs. Supporting Table 3: Comparison of c-REDUCE with Tree Gibbs Sampler. Supporting Table 4: Comparison of c-REDUCE with Converge and PhyloCon. Click here for file
